# Antiviral treatment in outpatients with herps zoster in six major areas of China, 2010–2019

**DOI:** 10.3389/fpubh.2022.942377

**Published:** 2022-07-29

**Authors:** Zhenwei Yu, Yuhua Zhao, Jiayi Jin, Jianping Zhu, Lingyan Yu, Gang Han

**Affiliations:** ^1^Sir Run Run Shaw Hospital, School of Medicine, Zhejiang University, Hangzhou, China; ^2^Affiliated Xiaoshan Hospital, Hangzhou Normal University, Hangzhou, China; ^3^Second Affiliated Hospital, School of Medicine, Zhejiang University, Hangzhou, China

**Keywords:** acyclovir, valaciclovir, famciclovir, prescription, cost

## Abstract

**Objective:**

The objective of this study was to assess the status and trends of antiviral treatment in outpatients with herpes zoster in China.

**Methods:**

Prescription data on antiviral drugs were extracted from the database of the Hospital Prescription Analysis Program of China according to the inclusion criteria. Yearly prescriptions and costs were calculated, and trends were analyzed. The trends were further stratified by age, sex, and specific drug use. The distribution of defined daily costs (DDCs) of valaciclovir and famciclovir were analyzed, and trends in the median DDCs were identified.

**Results:**

A total of 132,911 prescriptions from 49 hospitals located in six major areas of China were included in the analysis. The yearly prescriptions containing antivirals increased from 8,819 in 2010 to 16,361 in 2019. The percentage of prescriptions for patients aged 65 years and above also increased (27.7% in 2010 to 31.0% in 2019), and the number of prescriptions for females was higher than those for males (*P* < 0.001). The average cost of antivirals per prescription decreased; thus, the yearly cost showed no increasing trend. The main prescribed antivirals were valaciclovir and famciclovir, which progressively increased in prescriptions. The use of acyclovir decreased during the study period. Prescriptions containing topical formulations, acyclovir and penciclovir, both increased. The DDCs of valaciclovir and famciclovir decreased dramatically.

**Conclusion:**

The use of antivirals has increased over the decade, while the cost has not. Antiviral treatments adhere well to recent recommendations, except for the use of topical antivirals. The findings of this study may benefit the healthcare source allocation and management of herpes zoster in China.

## Introduction

Herpes zoster, also known as shingles, is an infection caused by latent varicella zoster virus reactivation and is usually characterized by a prodromal period with burning pain for two–three days, followed by a vesicular eruption in the dermatomal distribution of the infected ganglion (1). The global incidence of HZ ranges between 3–5 per 1,000 person-years (2). The incidence rate in China is similar, and it is estimated that at least 2.77 million cases occur in China annually (3, 4). It is also estimated that persons who live to 85 years of age have a 50% risk of herpes zoster in the absence of the herpes zoster vaccine (5).

Although most cases of herpes zoster are self-limited and will resolve within a few weeks, nearly all patients experience pain, with impact their normal functioning, reduces quality of life, and results in productivity losses. Herpes zoster is also associated with certain complications, the most common being post-herpetic neuralgia, a pain that persists long beyond cutaneous healing (1). Herpes zoster and its complications have been reported to cause approximately 67,000 quality-adjusted life years (QALYs) and incur costs of $2.4 billion in direct medical costs and productivity losses annually in the US (6). Economic studies in other countries and in China have also revealed that herpes zoster was associated with impaired quality of life and substantial health care resource use (7–10). Thus, attention should be paid to the management of herps zoster.

Antiviral therapy is recommended for all patients with herpes zoster, especially in patients with severe infection, old age, and immunocompromised status (1, 5, 11–13). The timely use of antiviral drugs, usually within 72 h of rash onset, can reduce viral replication, shorten the duration of symptoms, and prevent complications (1). Understanding the status of antiviral therapy for patients with herpes zoster would be helpful for improving disease management. However, little is known about this issue, especially in China. Thus, we conducted this cross-sectional study to assess the patterns and trends of antiviral therapy for patients with herpes zoster over the past decade.

## Methods

### Study design and ethics

This study was designed as a database-based, cross-sectional study. Ethical approval was obtained from the Ethics Committee of Sir Run Run Shaw Hospital, College of Medicine, Zhejiang University (Reference Number 20210924-33). Informed consent was waived because of the retrospective nature of the study.

### Data collection

Data on prescriptions were obtained from the Hospital Prescription Analysis Cooperative Project of China, which is widely used in pharmacoepidemiology studies (14–19). Prescriptions that met the following criteria were included for analysis: (1) Prescriptions from hospitals located in six major area of China (Beijing, Shanghai, Guangzhou, Chengdu, Hangzhou and Tianjin); (2) Prescriptions from hospitals that participated the program continuously, from 2010 to 2019; (3) Prescriptions written during 2010 and 2019; (4) Prescriptions written for adult outpatients (aged 18 years and above) with the diagnosis of herps zoster; (5) Prescriptions containing at least one antiviral drugs. The antiviral drugs were limited to acyclovir (J05AB01 and D06BB03), valacyclovir (J05AB11), famciclovir (J05AB09), penciclovir (D06BB06) and ganciclovir (J05AB06) in this study.

### Analysis

The yearly prescription numbers of antiviral drugs for patients with herpes zoster were represented by the yearly eligible prescription numbers. The corresponding yearly cost was obtained by adding up the cost of all antiviral drugs in that year. It should be noted that this study did not extrapolate sampled data. Trends in yearly prescriptions and costs were analyzed and further stratified by specific drugs.

The percentages of valaciclovir (or famciclovir) prescriptions were evaluated at the hospital level. They were obtained by dividing the yearly prescriptions that contained valaciclovir (or famciclovir) by the total yearly eligible prescriptions in specific hospitals, and the yearly distribution of percentages was represented by a violin plot.

The defined daily cost (DDC), also known as the cost of a defined daily dose, was studied at the prescription level. DDCs were calculated for specific prescriptions using the following equation, and their distribution was also analyzed.


(1) $$\begin{array}{r} \text{Defined daily cost}=\frac{price\;of\;drug}{strength\;of\;price\;unit/defined\;daily\;dose} \end{array}$$

The trends in the identified values were tested using the Mann–Kendal test, and the trends in percentages were tested using a log-linear test. The differences in the percentages of prescriptions for males and females were tested using the chi-square test. Statistical analysis was performed using R software. Statistical significance was set at *p* < 0.05.

## Results

### Demographic characteristics of prescriptions and overall trends

**Figure 1:**
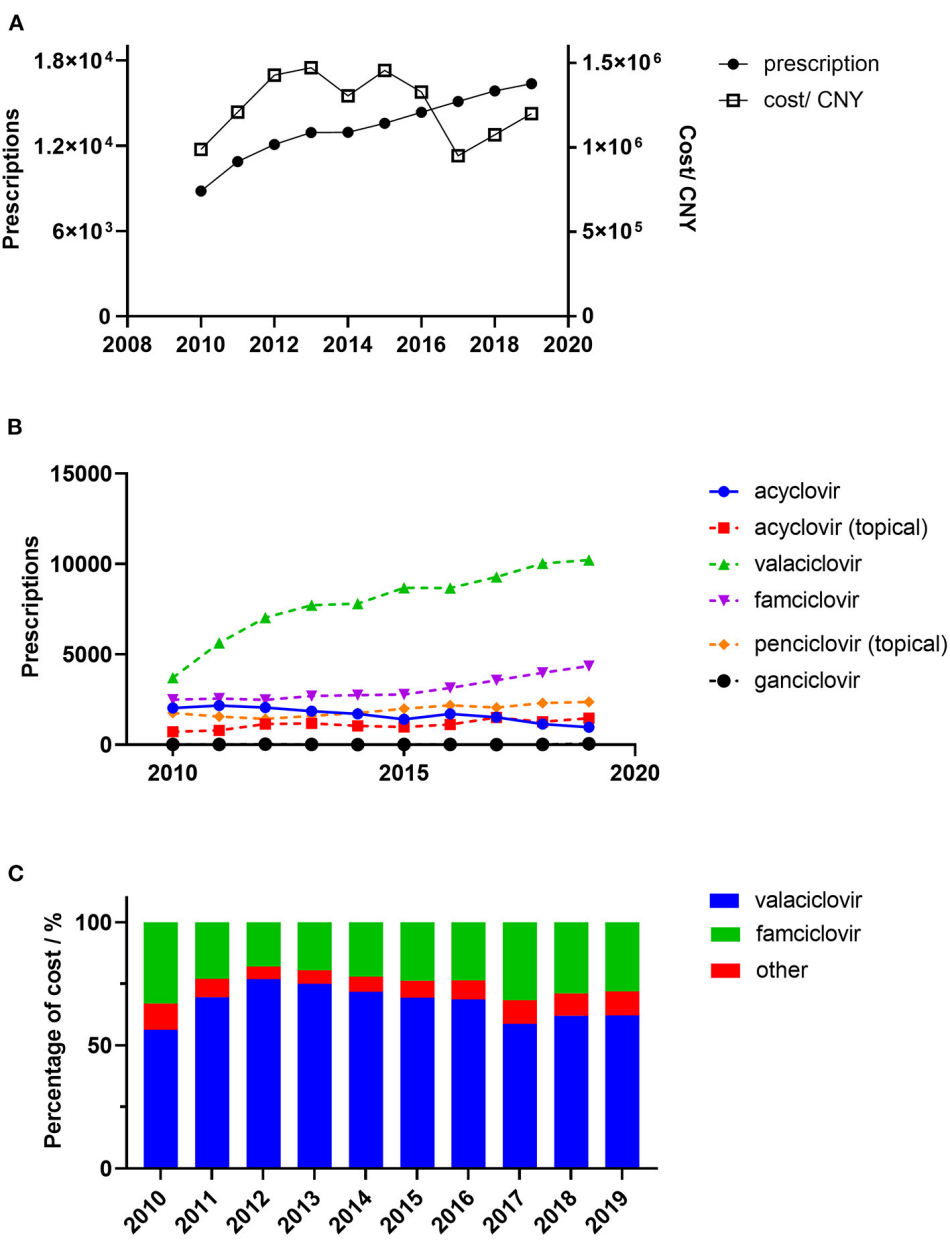
Trends in the prescription of antiviral drugs for patients with herps zoster in six major areas of China. **(A)** Overall yearly prescriptions and cost; **(B)** Yearly prescription of individual drugs; **(C)** Antiviral cost percentages. Percentage of cost means the percentage of overall yearly cost of specific drug.

A total of 132,911 prescriptions from 49 hospitals were included in this study. The yearly prescriptions and corresponding costs are shown in Figure 1A. Yearly prescriptions increased progressively from 8,819 in 2010 to 16361 in 2019 (*P* < 0.001). The average cost per prescription is shown in Table 1, which reveals a significant decreasing trend over the study period (*P* = 0.012). Thus, the yearly cost fluctuated over the decade and showed no significant trend (*P* = 0.721). The demographic characteristics of patients with antiviral prescriptions are shown in Table 1. The percentage of prescriptions for females and males did not change over the study period (*P* = 0.737). However, the number of prescriptions for females was higher than those for males in each year (all *P* < 0.05). The majority of prescriptions were for patients aged 45–64 years, and, in this age range, the percentage of prescriptions remained constant (*P* = 0.364). However, the percentage of prescriptions for patients aged 65 years and above increased progressively (*P* = 0.019).

**Table 1:** Characteristics of patients with antiviral prescriptions and average cost per prescription.

		**2010**	**2011**	**2012**	**2013**	**2014**	**2015**	**2016**	**2017**	**2018**	**2019**
Age (year)	18–44	2,630 (29.8)	3,214 (29.5)	3,795 (31.4)	3,939 (30.5)	3,788 (29.3)	4,018 (29.6)	4,198 (29.3)	4,331 (28.7)	4,549 (28.7)	4,554 (27.8)
	45–64	3,745 (42.5)	4,628 (42.5)	5,067 (41.9)	5,469 (42.3)	5,640 (43.6)	5,825 (42.9)	6,230 (43.4)	6,389 (42.3)	6,629 (41.8)	6,729 (41.1)
	≥65	2,444 (27.7)	3,050 (28.0)	3,221 (26.7)	3,512 (27.2)	3,519 (27.2)	3,739 (27.5)	3,918 (27.3)	4,387 (29.0)	4,676 (29.5)	5,078 (31.0)
Sex	Male	3,992 (45.3)	4,957 (45.5)	5,647 (46.7)	6,020 (46.6)	5,882 (45.4)	6,170 (45.4)	6,564 (45.8)	6,985 (46.2)	7,188 (45.3)	7,455 (45.6)
	Female	4,827 (54.7)	5,935 (54.5)	6,436 (53.3)	6,900 (53.4)	7,065 (54.6)	7,412 (54.6)	7,782 (54.2)	8,122 (53.8)	8,666 (54.7)	8,906 (54.4)
Average cost (CNY)		112.1 ± 91.0	111 ± 91.2	118.2 ± 117.7	114 ± 144.6	100.9 ± 95.5	107.3 ± 111.6	92.6 ± 95.0	62.9 ± 87.2	67.8 ± 92.1	73.4 ± 103.9

*Data were presented as prescription numbers (percentage%). Average cost was presented as mean ± standard deviation in CNY*.

### Trends in the prescriptions of specific antiviral drugs

**Figure 2:**
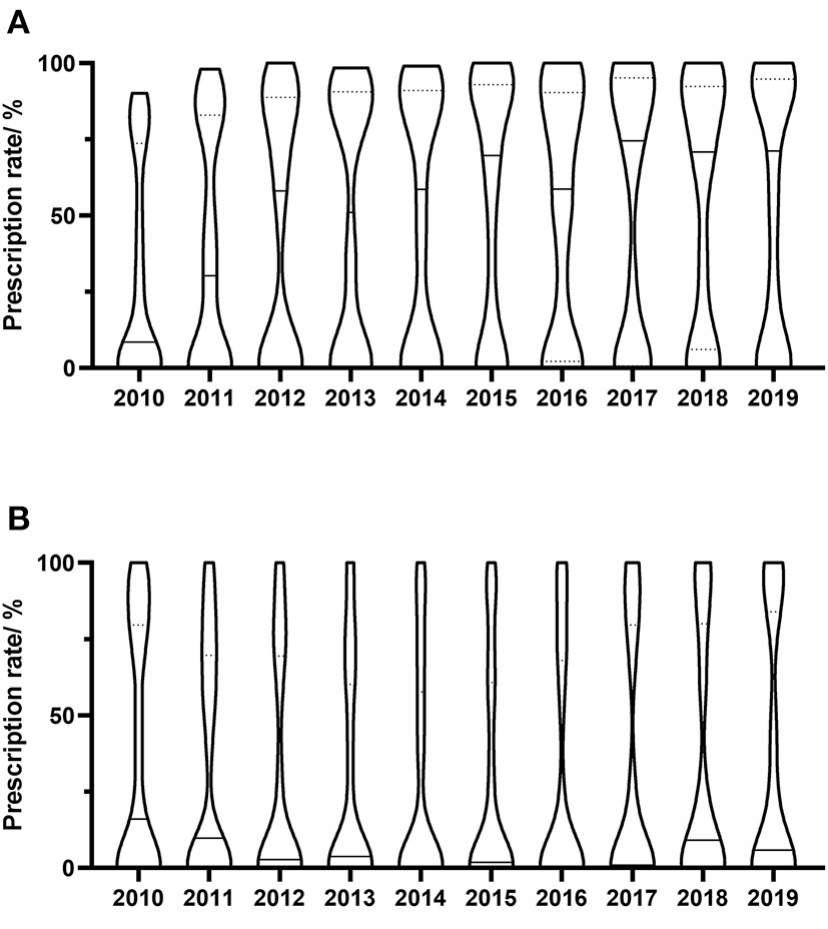
Yearly distribution of antivirals prescriptions in individual hospitals. **(A)** Valaciclovir; **(B)** Famciclovir. Prescription rate means the yearly percentage of prescriptions containing specific drug. Each column shows the distribution of prescription rate of prescriptions containing specific drug, and the width of the column represents the relative number of hospitals. The dotted lines in column represent the first quartile and the third quartile of the distribution, and the solid line in column represents the median of the distribution.

The yearly prescriptions of each antiviral drug are shown in Figure 1. Valaciclovir and famciclovir were the major prescribed antiviral drugs over all of the years. Both of their prescriptions increased progressively during the 10 years (both *P* < 0.001), while prescriptions that contained acyclovir decreased over the study period (*P* = 0.002). The percentages of prescriptions that contained valaciclovir and famciclovir in individual hospitals were calculated, and their yearly distributions are shown in Figure 2. Our findings indicate that the treatment patterns differed greatly among hospitals. Some hospitals prescribe valaciclovir to most patients, while others prescribe valacyclovir to a very small percentage of patients.

Ganciclovir was rarely used, and its prescription was stable (*P* = 0.530). There were also two topical formulations, acyclovir and penciclovir, both of which showed increasing trends in prescription numbers over the study period (*P* = 0.020 and *P* = 0.002, respectively).

### Trends in cost of specific drugs

**Figure 3:**
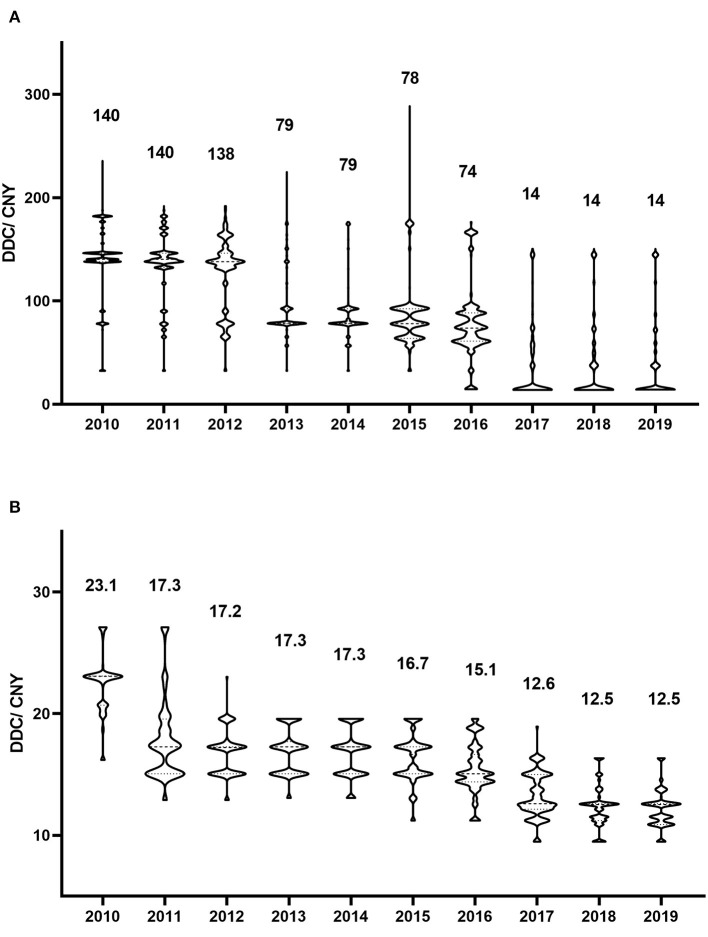
Defined daily cost of antivirals in each year. **(A)** Valaciclovir; **(B)** Famciclovir. The numbers represent the median defined daily cost for each year.

From a cost perspective, valaciclovir and famciclovir were the major prescribed antiviral drugs (Figure 1C). However, the cost shares showed no significant trends for either drug (*P* = 0.447 and *P* = 0.345, respectively). The distribution of valaciclovir and famciclovir DDCs in each year is shown in Figure 3. There were dramatic decreases in the DDCs of valaciclovir and famciclovir (as shown by the trend in yearly median DDC, *P* < 0.001 and *P* = 0.001, respectively). The median DDC of valaciclovir in 2019 was approximately one-tenth of that in 2010, and the median DDC of famciclovir in 2019 was approximately half of that in 2010. The percentage of the other four antiviral drugs was <10% of the total cost and did not change during the study period (Figure 1C, *P* = 0.340).

## Discussion

To the best of our knowledge, this is the first study to assess the patterns and trends of antiviral therapy in patients with herpes zoster in China. The yearly prescriptions containing antiviral drugs for patients with herpes zoster have been increasing progressively. The main prescribed antiviral drugs during our study period were valacyclovir and famciclovir. The DDCs of valaciclovir and famciclovir decreased progressively over the study period, and the yearly cost was stable.

The increase in yearly antiviral prescriptions may be due to the increased incidence of herpes zoster. A temporal increase in the incidence of herpes zoster has been reported over the past several decades across several countries (2). A recent study concluded that herpes zoster incidence rates in the US population increased in all age groups from 1991 to 2016 (20). Data on trends in the incidence rate of herpes zoster in China are limited. A recent study reported that the overall incidence of herpes zoster in the population aged over 50 years in China was 6.64 per 1000 person-years (10). Another possible reason for the increased prescription of antivirals could be the increasing demand for better management of herpes zoster in China. It has been reported that only 58% of patients with herpes zoster received antivirals in the UK (21). This may be due to a lack of confidence in antivirals and delayed treatment. The population of outpatients receiving oral antivirals in the US was also near 64%, (22) which is similar to the percentage of Chinese patients receiving antivirals (10). Oral antiviral medications were found to be effective in reducing the duration of acute neuritis symptoms and the risk of complications. Our findings indicate that efforts should be made to increase the percentage of patients receiving antiviral treatment.

Age is widely acknowledged as an independent risk factor for herpes zoster (3, 10). In our study, prescriptions for patients aged <45 years only accounted for a small proportion, and the percentage of patients for this age range continues to decrease. Notably, prescriptions for patients aged over 65 years increased progressively during our study period. Routine vaccination for individuals over 60 years has shown considerable effect in terms of reducing the incidence of herpes zoster, but a recent study showed that the vaccination willingness was only 16.6% in Chinese aged 50-69 years (23, 24). Elderly patients are more likely to benefit from antiviral therapy, however, older adults are also more likely to develop drug adverse events (25). Moreover, the studied antivirals mainly undergo renal excretion and should be closely monitored in patients with reduced renal clearance, which is common in elderly patients (11, 26). The number of prescriptions for females was greater than those for males, which is in accordance with epidemiological findings of female sex being more significantly associated with herpes zoster (3, 10).

As this study focused on outpatients, and only oral and topical antivirals were analyzed. Certain novel antivirals, such as brivudine, are not usually suggested or commonly supplied in Chinese hospitals nor were they included in this study analysis. In this study, the overall use of valaciclovir was greater than that of famciclovir. Moreover, the use of these two agents differed greatly among hospitals. A literature report on antiviral drug use in the district of an eastern city of China during 2015 and 2017 found that acyclovir was the major antiviral drug (10). When looking at the situation in other countries, the use of antivirals also differed greatly. A study in the UK found that major antiviral agent was acyclovir in a period of 2000–2011 (21). However, the major antiviral prescribed to US outpatients was valaciclovir, followed by acyclovir and famciclovir (22). A survey of German primary physicians revealed that famciclovir and valaciclovir were the most commonly prescribed drugs (27). Interestingly, acyclovir was found to be the only antiviral drug prescribed to patients with herpes zoster in a New Zealand study (28).

Oral valaciclovir, famciclovir, and acyclovir are all recommended as they are proven to be superior to placebo in reducing the amount of time to complete cessation of pain (1). Valaciclovir is a prodrug of acyclovir with high bioavailability, and famciclovir is the prodrug of penciclovir. There were no differences in cutaneous and pain endpoints between famciclovir and valaciclovir (1). A recent randomized trial showed that famciclovir was not superior to acyclovir in the efficacy of herpes zoster treatment in adults (29). Furthermore, the three antivirals were found to have similar efficacy and safety profiles (1, 29). Valacyclovir and famciclovir are preferable to acyclovir because of their better pharmacokinetic profiles, easier dosing schedules, and probable higher levels of antiviral drug activity (5, 12). Given the prescription trends, it seems that physicians in China follow the guidelines well.

We found that treatment patterns differed greatly among hospitals. Some hospitals prescribe valaciclovir to most patients, while others prescribe valacyclovir to a very small percentage of patients. A similar situation was found with the use of famciclovir. The choice of valaciclovir or famciclovir may be affected by physician prescription habits, drug supply, and accessibility; however, either is reasonable, especially at the end of this study, when the DDCs of the two agents were found to be similar.

There is insufficient evidence to support the use of topical treatment for acute herpes zoster, and topical antivirals are not recommended in various expert opinions and guidelines (1, 11, 12). However, the prescription of the two topical antiviral formulations is increasing.

Cost is another important factor that influences the choice of antiviral therapy by physicians and patients. The health insurance system can also be a concern, and it is interesting to note that the overall cost of antivirals did not increase, despite the progressive increase in prescriptions, during our study period. The share in cost of valaciclovir or famciclovir did not change with their increasing use, which was due to a dramatic decrease in the DDCs of valaciclovir and famciclovir. Valaciclovir was expensive at the beginning of the study, but the median DDC in 2019 was approximately one-tenth of that in 2010. The reason for this was the implementation of the volume-based purchasing program and the national centralized drug purchasing pilot program in China (30–32). Many countries are facing the challenge of ever-increasing pharmaceutical expenditures, and it is common practice worldwide that lowering drug prices and reducing drug expenditures by volume-based drug procurement (31, 33, 34). Our results indicated that these programs reduced drug expenditure, increased the use of policy-related drugs, and may increase drug accessibility.

This study has some limitations. First, the sampling hospitals were located in six major areas of China, which may have resulted in a sampling bias. Furthermore, the severity of herpes zoster, as well as the clinical outcomes of antiviral treatment, have not been well studied. Other commonly co-used drugs for patients with herpes zoster, such as analgesics, should be evaluated in future studies.

## Conclusion

We assessed the status and trends of antiviral therapy in outpatients with herpes zoster in China over a decade. During the study period, the prescription of antiviral drugs increased progressively. The main prescribed antivirals were valaciclovir and famciclovir, but the use of these two differed greatly among hospitals. Acyclovir showed a decreasing trend in prescriptions. Topical antivirals are not recommended, however, their use continues to increase. The yearly cost remained stable, due to the decreasing DDCs of valaciclovir and famciclovir. The antiviral treatments adhere well to recent recommendations, except for the use of topical antivirals. The findings of this study may benefit the healthcare source allocation and management of herpes zoster in Chinese outpatients.

## Data availability statement

The original contributions presented in the study are included in the article/Supplementary material, further inquiries can be directed to the corresponding authors.

## Ethics statement

The studies involving human participants were reviewed and approved by Ethics Committee of Sir Run Run Shaw Hospital, College of Medicine, Zhejiang University. Written informed consent for participation was not required for this study in accordance with the national legislation and the institutional requirements.

## Author contributions

Conceptualization, project administration, supervision, validation, writing—review and editing: LY and GH. Data curation: ZY, YZ, JJ, and JZ. Formal analysis and investigation: ZY and YZ. Methodology: ZY, LY, and GH. Resources, software, visualization, and roles/writing—original draft: ZY. All authors contributed to the article and approved the submitted version.

## Conflict of interest

The authors declare that the research was conducted in the absence of any commercial or financial relationships that could be construed as a potential conflict of interest.

## Publisher's note

All claims expressed in this article are solely those of the authors and do not necessarily represent those of their affiliated organizations, or those of the publisher, the editors and the reviewers. Any product that may be evaluated in this article, or claim that may be made by its manufacturer, is not guaranteed or endorsed by the publisher.
