# Supplementary figures and images for: Antiviral treatment in outpatients with herps zoster in six major areas of China, 2010–2019
Source: Front Public Health. 2022 Jul 29;10:942377. doi: 10.3389/fpubh.2022.942377 (PMC9372588; doi:10.3389/fpubh.2022.942377)

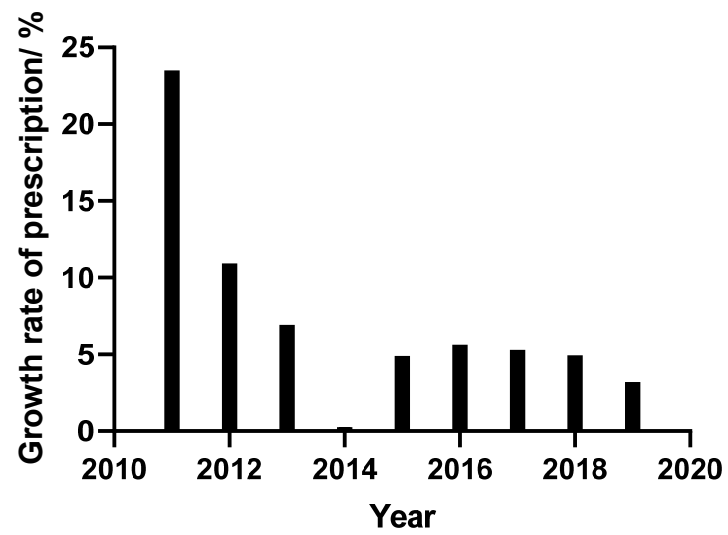

Fig. S1 Growth rate of prescription in each year

Supplement: Supplementary file 1 [file Data_Sheet_1.PDF]
